# The Impact of Concomitant Upper Extremity Fractures on Outcomes in Geriatric Patients Following Hip Fracture Surgery

**DOI:** 10.3390/jcm14186380

**Published:** 2025-09-10

**Authors:** Nadav Graif, Etay Elbaz, Yaniv Warschawski, Efi Kazum, Lior Shabtai, Nissan Amzallag, Shai Factor

**Affiliations:** 1 Department of Orthopedic Surgery, Tel Aviv Medical Center, Gray Faculty of Medical & Health Sciences, Tel Aviv University, Tel Aviv 69978, Israel; 2 Zucker School of Medicine at Hofstra/Northwell, Hempstead, NY 11549, USA

**Keywords:** hip fracture, upper extremity fractur, geriatric, length of stay, mortality

## Abstract

**Background:** Hip fractures in geriatric patients represent a major public health burden, with a clinically important subset presenting with concomitant upper extremity (UE) fractures. The independent impact of these dual injuries on clinical outcomes remains incompletely characterized. **Methods:** A retrospective study of patients aged ≥65 years who underwent surgical treatment for hip fracture at tertiary medical center, between January 2010 and January 2024. Patients were stratified based on the presence of a UE fracture sustained at the same time as the hip fracture. Multivariable regression models were used to assess outcomes, adjusting for age, sex, hip fracture type, and comorbidity burden. Primary outcomes were hospital length of stay and mortality at 30 days and 1 year. Secondary outcomes included readmission rates, revision surgery, and infection complications. **Results:** Of 7488 patients, 251 (3.4%) had concomitant upper extremity (UE) fractures. These patients had a longer mean hospital stay compared with isolated hip fractures (20.2 vs. 17.5 days, *p* = 0.047), with no significant difference in 30-day mortality (*p* = 0.439) and a trend toward lower 1-year mortality (*p* = 0.058). In the concomitant UE fracture group, operative treatment was associated with longer hospitalization (26.2 vs. 19.2 days, *p* = 0.05) and higher revision surgery rates (14.0% vs. 3.1%, *p* = 0.01). Subgroup analyses by fracture type showed similar trends, with longer hospital stays observed in intracapsular fractures with concomitant injury (*p* = 0.05). Subgroup analysis by UE fracture location showed significantly longer stays for distal radius fractures compared with isolated hip fractures, whereas no significant differences were observed for proximal humerus or other UE fracture locations. **Conclusions:** Concomitant UE fractures in geriatric hip fracture patients are associated with prolonged hospitalization. Operative management of UE fractures results in longer hospital stays and an increased risk of revision surgery. These findings highlight the importance of tailored perioperative planning and resource allocation for this vulnerable patient group.

## 1. Introduction

Hip fractures in geriatric patients represent one of the most significant healthcare challenges of the 21st century, affecting over 300,000 individuals annually in the United States alone, with projected increases as the population ages [1]. Globally, hip fractures constitute a worldwide epidemic affecting an estimated 14.2 million individuals annually, with substantial geographic variation in incidence rates. Age- and sex-standardized incidence rates range from 95.1 per 100,000 population in Brazil to 315.9 per 100,000 in Denmark, while in European countries, the lifetime probability of hip fracture at age 50 varies from 7.0–25.1% in women and 3.8–10.9% in men. Asian populations demonstrate significantly lower incidence rates compared to Western populations [2]. The associated morbidity and mortality remain substantial, with 1-year mortality rates ranging from 20% to 30%. Among those who survive, a substantial proportion do not regain their pre-fracture level of functional independence [3,4].

A clinically important subset of geriatric hip fracture patients, estimated to affect 3–8% of cases, sustains concurrent upper extremity (UE) fractures [5,6]. This combination presents unique clinical challenges, as hip fractures severely compromise lower extremity mobility while UE injuries can significantly impair the ability to use assistive devices, potentially complicating rehabilitation and delaying recovery. In addition, the presence of UE fractures may necessitate modifications in perioperative positioning, anaesthesia management, and physical therapy protocols, placing additional strain on both patients and healthcare systems [7,8].

Despite the clinical importance of these dual injuries, the precise impact of concomitant UE fractures on clinical outcomes remains controversial. Previous studies have reported conflicting findings, with some suggesting increased mortality and complications [9,10], while others found no significant differences [11]. This ambiguity is compounded by methodological limitations including small sample sizes, inadequate adjustment for confounding variables, and heterogeneous outcome measures. Moreover, many prior investigations have not stratified outcomes by fracture location, surgical versus non-surgical management, or hip fracture type, limiting the applicability of their conclusions to individualized patient care. Key evidence gaps include a lack of data on time-dependent outcomes, limited application of advanced statistical matching techniques to mitigate selection bias, and insufficiently detailed analyses of specific UE fracture patterns and their management. Importantly, resolving these uncertainties is critical for clinical practice, as it can directly inform treatment decisions, surgical planning, and risk stratification for geriatric patients with concomitant injuries.

The primary objectives of this study were to determine the independent impact of concomitant UE fractures on hospital length of stay and mortality in geriatric hip fracture patients, with detailed subgroup analyses examining the effects of fracture location, treatment approach, and hip fracture type. Secondary objectives included evaluation of hospital readmission, revision surgery and the occurrence of infections (surgical and non-surgical) at predefined time points. By providing a comprehensive assessment using a large, contemporary cohort, this study aims to inform evidence-based strategies for the management of complex hip fracture cases with concomitant UE injuries. This focus on clinically relevant outcomes ensures that the findings can guide real-world decision-making and improve patient care.

## 2. Methods

### 2.1. Study Design and Population

A retrospective cohort study was conducted using electronic medical records from a Level 1 trauma center serving a large metropolitan population. Following approval from the Institutional Review Board, all patients who were admitted with a primary diagnosis of hip fracture requiring surgical intervention between 1 January 2010, and 31 January 2024, were identified. Inclusion criteria were: (1) age ≥ 65 years, (2) primary hip fracture requiring surgical intervention, (3) complete demographic data, and (4) minimum 1-year follow-up or death within the study period. Exclusion criteria included: (1) pathological fractures, (2) periprosthetic fractures, (3) patients transferred from other institutions with incomplete initial data, and (4) pre-existing non-ambulatory status. All patient data were retrospectively collected from electronic medical records (EMR), which are fully digitized and meticulously maintained at our institution. This system allowed for comprehensive retrieval of demographic information, fracture characteristics, treatment details, and outcomes, including hospital length of stay, revision surgeries, infections, and mortality. Data abstraction was performed systematically to ensure accuracy and completeness across the cohort.

The main exposure variable was the presence of a concomitant UE fracture, defined as any radiographically confirmed fracture of the humerus, radius, ulna, or hand bones sustained during the same injury event as the hip fracture.

The primary outcomes included hospital length of stay and all-cause mortality at 30 days, 90 days, and 1 year following surgery. Secondary outcomes comprised all-cause hospital readmission at 7-, 30-, and 90-days post-discharge; revision surgery of the index hip within 1 year; and the occurrence of infections. Infections were categorized as surgical site infections (superficial or deep) and non-surgical infections (including urinary tract infections, pneumonia, and bacteraemia), assessed at 30 days, 90 days, and 1 year.

Covariates included patient age, sex, body mass index (BMI), American Society of Anaesthesiologists (ASA) physical status score, Charlson Comorbidity Index (CCI), hip fracture type (intracapsular vs. extracapsular), and specific comorbidities including diabetes mellitus, congestive heart failure, chronic obstructive pulmonary disease, and renal disease.

### 2.2. Statistical Analysis

Descriptive statistics were calculated for all variables. Between-group comparisons used independent *t*-tests for continuous variables and chi-square or Fisher’s exact tests for categorical variables. Multivariable regression models were used to determine whether concomitant UE fractures represent an independent risk factor for adverse outcomes after controlling for potential confounding variables. The primary objective was to establish whether the presence of concomitant fractures independently predicts clinical outcomes beyond the effects of age, comorbidity, and other patient characteristics. A multivariable logistic regression model estimated the probability of having a concomitant UE fracture based on clinically relevant covariates: age (continuous), sex, hip fracture type, CCI score (continuous), and key comorbidities. Subgroup Analysis were performed for: (1) intracapsular vs. extracapsular hip fractures, (2) UE fracture location (distal radius vs. proximal humerus vs. other), and (3) treatment approach (operative vs. non-operative management of concomitant fractures). The final cohort included 7488 patients, of whom 251 (3.4%) sustained concomitant upper extremity fractures. Although no formal a priori sample size calculation was performed due to the retrospective design, the large cohort size allows sufficient statistical power to detect clinically meaningful differences in primary outcomes, including hospital length of stay and 1-year mortality. Post hoc power estimates based on the observed effect sizes and outcome variability confirm that the study was adequately powered to evaluate these associations. Statistical significance was set at *p* < 0.05 (two-tailed). All analyses were performed using R version 4.4.0.

## 3. Results

### 3.1. Study Cohort and Baseline Characteristics

Of the 7545 patients initially screened, 57 were excluded based on age < 65 years, resulting in a final cohort of 7488 patients. Among these, 7237 (96.6%) had isolated hip fractures, while 251 (3.4%) sustained concomitant UE fractures. Among patients with concomitant fractures, the most common locations were distal radius (114 patients, 45.4%) and proximal humerus (103 patients, 41%). Prior to multivariable adjustment, statistically significant differences were observed between the groups (Table 1). The concomitant fracture group had a higher proportion of female patients (84.1% vs. 66.9%, *p* < 0.001), different comorbidity distribution (CCI *p* = 0.002), and different hip fracture type distribution (34.7% vs. 39.7% intracapsular, *p* = 0.050)). No significant differences were observed in age, body mass index, or ASA classification.

### 3.2. Primary Outcomes

In multivariable analysis, concomitant UE fractures were independently associated with significantly longer hospital stays after adjusting for age, sex, hip fracture type, and comorbidity burden (adjusted difference +2.7 days, 95% CI: 0.1–5.3, *p* = 0.047). No significant difference in 30-day mortality was observed between groups (3.2% vs. 4.2%, 95% CI: 0.37–1.54 *p* = 0.439), while a trend toward lower 1-year mortality was noted in patients with concomitant UE fractures (12.4% vs. 17.0%, 95% CI: 0.47–1.01 *p* = 0.058).

### 3.3. Secondary Outcomes

Patients with concomitant fractures had higher rates of 7-day readmission (3.2% vs. 1.3%, 95% CI: 1.19–5.29 *p* = 0.016), however, 30-day readmission rates were not significantly different between groups (7.2% vs. 6.0%, 95% CI: 0.75–1.99, *p* = 0.426). Revision surgery rates at 1 year were not significantly different overall (adjusted OR 1.29, 95% CI: 0.75–2.22, *p* = 0.356), though this finding varied significantly by treatment approach for the concomitant fracture. Infection rates at 1 year were similar between groups for both surgical site infections (4.8% vs. 4.1%,95% CI: 0.72–1.93, *p* = 0.507) and non-surgical infections (15.5% vs. 15.1%, 95% CI: 0.74–1.43 *p* = 0.864) (Table 2).

### 3.4. Subgroup Analyses

When stratified by fracture location, distal radius fractures demonstrated the strongest association with prolonged hospitalization, with an adjusted increase of +4.0 days compared to isolated hip fractures (*p* = 0.05). Proximal humerus fractures were associated with a non-significant increase in length of stay (+2.4 days, *p* = 0.10), whereas other upper extremity fracture locations showed no difference (–0.3 days, *p* = 0.50). Distal radius fractures were also associated with lower adjusted 1-year mortality compared with isolated hip fractures (7.9% vs. 17.0%; adjusted OR 0.44, *p* = 0.05). Within one year of fracture, there were no significant differences across fracture locations in revision surgery (distal radius 6.1%, proximal humerus 5.8%, other upper extremity locations 2.9%; *p* = 0.8), incidence of any infection (distal radius 16.7%, proximal humerus 20.4%, other upper extremity locations 8.8%; *p* = 0.46), or 90-day readmission rates (distal radius 11.4%, proximal humerus 10.7%, other upper extremity locations 8.8%; *p* = 0.95). These findings suggest comparable short- and medium-term complication rates across fracture groups beyond the primary outcomes of LOS and mortalit (Table 3).

When outcomes were analyzed according to treatment strategy, operative fixation of concomitant upper extremity fractures was associated with substantially worse short- and long-term outcomes compared with non-operative management. Upper extremity procedures were performed on average 3 days after the hip fracture surgery (range 0–10 days). Patients who underwent operative treatment had a significantly longer hospital stay (adjusted +8.6 vs. +1.3 days relative to isolated hip fractures, *p* = 0.05) and a markedly higher risk of revision surgery within 1 year (adjusted OR 4.02, *p* = 0.05). One-year mortality was lower in the operative group (5.3% vs. 14.4%), although this difference did not reach statistical significance (*p* = 0.07). No significant differences were observed in infection or 90-day readmission rates between the groups (Table 4).

When stratified by hip fracture type, the impact of concomitant upper extremity fractures was more pronounced among patients with intracapsular hip fractures. These patients had significantly prolonged hospitalizations (adjusted +5.5 days, *p* = 0.05) compared with isolated intracapsular fractures, whereas the difference was smaller and not statistically significant in extracapsular fractures (adjusted +1.5 days, *p* = 0.10). No differences were observed in 30-day mortality for either fracture type. At 1-year, extracapsular fractures with concomitant injuries were associated with a modestly lower adjusted mortality risk (adjusted OR 0.62, *p* = 0.05), while no significant difference was seen in the intracapsular group (Table 5).

## 4. Discussion

This large, analysis of 7488 geriatric hip fracture patients demonstrates that concomitant UE fractures independently predict significantly prolonged hospital stays and increased early readmission risk but are not associated with increased mortality.

Contrary to previous reports [9,10], this study found no association between concomitant fractures and increased mortality risk. When critical factors such as age, comorbidities, and fracture characteristics are adequately controlled, additional limb fractures may not independently influence mortality. This finding underscores the importance of comprehensive risk adjustment in studies of geriatric trauma populations, as unadjusted analyses may overestimate the prognostic impact of concomitant injuries [11]. The primary determinants of survival in this vulnerable population appear to be underlying physiological reserve and pre-injury health status rather than specific fracture patterns. Moreover, these results suggest that clinical focus should prioritize optimization of overall medical status, perioperative management, and prevention of systemic complications, rather than concentrating solely on the management of additional fractures.

The 2.7-day increase in mean hospital length of stay represents both a clinically meaningful and economically significant burden [12]. For healthcare systems, this translates to significant resource utilization requiring consideration in capacity planning and budget allocation. This prolongation likely reflects the compounding functional impairment resulting from injuries to both upper and lower extremities, which severely complicates mobility, self-care activities, and safe discharge planning. Importantly, this effect appears most pronounced in patients with intracapsular hip fractures and those receiving operative management of their UE injuries.

Subgroup analysis revealed that operative management of concomitant fractures was associated with a three-fold increase in revision rates and substantially longer hospitalization, compared to non-operative treatment. The decision to pursue operative versus non-operative management should consider not only fracture characteristics but also the patient’s overall medical status, rehabilitation potential, and the substantial impact on hospital resource utilization. This observation aligns with concerns about the increased surgical burden and complexity in this frail geriatric population. While surgical fixation of UE fractures may facilitate anatomical alignment and potentially expedite functional recovery, it concurrently exposes patients to elevated risks of postoperative complications such as infections, hardware failure, and anaesthesia-related adverse events, which can necessitate revision procedures. Moreover, the cumulative physiological stress of undergoing two major procedures in close succession, hip fracture surgery followed by UE fixation, may further compromise postoperative recovery and contribute to delayed mobilization. Previous studies focusing specifically on geriatric patients with combined hip and UE fractures remain limited but suggest a nuanced balance between the benefits and risks of surgical intervention [13]. Consequently, individualized decision-making, integrating geriatric, anaesthetic, and rehabilitation perspectives, is essential to determine whether the theoretical benefits of fixation outweigh the substantial risks in this high-risk cohort.

Contrary to previous reports suggesting differential outcomes based on the anatomical location of UE fractures, such as Jodoin et al. [10], who reported worse in patients with proximal UE fractures compared to distal ones, and Morris et al. [14], who observed significantly higher inpatient and one-year mortality among patients with concurrent humerus fractures relative to distal radius fractures, the present study did not demonstrate significant differences in clinical outcomes between distal radius and proximal humerus fractures. These differences may be explained by several factors, including variations in cohort characteristics (e.g., age distribution, comorbidities), differences in treatment protocols (operative vs. non-operative management), study design, or sample size. In particular, our large, contemporary cohort and standardized management approach may have mitigated some of the risks observed in earlier, smaller or more heterogeneous populations. This suggests that, in certain populations, the anatomical location of the UE fracture may not independently influence outcomes. Rather, it may be the overall functional impairment resulting from any upper limb fracture, regardless of its specific location, that primarily contributes to prolonged hospitalization in geriatric hip fracture patients [15]. Recognizing this can guide clinicians to focus on optimizing overall functional support and rehabilitation, rather than targeting interventions based solely on fracture location. Impaired upper limb strength and coordination may limit the effective use of assistive devices, thereby delaying ambulation, prolonging rehabilitation, and increasing the risk of postoperative complications, as demonstrated by the association between greater grip strength and improved early mobility outcomes following hip surgery [16].

Overall, these findings support the development of specialized clinical pathways for patients with dual injuries, including early involvement of occupational therapy, specialized assistive devices, modified rehabilitation protocols, and enhanced discharge planning to optimize functional recovery. The higher revision rates associated with operative management of UE fractures emphasize the need for careful patient selection and shared decision-making regarding surgical intervention. Functional outcomes and rehabilitation potential, though not captured in this study, are critical considerations in patients with combined hip and upper extremity fractures. The compounded impairment from injuries affecting both mobility and upper limb function may substantially delay rehabilitation and impact quality of life. Future studies incorporating detailed functional assessments and patient-reported outcomes are needed to better understand the full clinical implications of concomitant UE fractures in this population.

## 5. Limitations

This study has several important limitations that should be considered. As a retrospective single-center study, generalizability may be limited. The study is susceptible to unmeasured confounders including pre-fracture functional status, cognitive function, and social support systems. Long-term functional outcome data were not available, limiting understanding of recovery patterns. A notable limitation of this analysis is the non-normal distribution of certain continuous variables, particularly length of stay, as evidenced by standard deviations exceeding the mean. This skewed distribution reflects substantial variability and may reduce the precision of mean-based comparisons, even when appropriate non-parametric tests were applied. The relatively small number of patients with specific concomitant fracture types limited power for some subgroup analyses. Finally, although our dataset was comprehensive, we cannot account for patients who may have been readmitted or sought treatment at other hospitals, and such events would not be captured in our institutional records. This may lead to underestimation of certain outcomes, such as complications or readmissions.

## 6. Conclusions

Concomitant UE fractures in geriatric hip fracture patients are associated with prolonged hospitalization. Operative management of UE fractures results in longer hospital stays and an increased risk of revision surgery. These findings highlight the importance of tailored perioperative planning and conservative treatment strategies to optimize functional recovery, discharge planning, and resource allocation in this vulnerable patient group.

## Figures and Tables

**Table 1:** Baseline characteristics.

Characteristic	Isolated Hip Fracture (n = 7237)	Concomitant Upper Extremity Fracture (n = 251)	*p*-Value
Age (years), mean ± SD	82.1 ± 8.2	82.6 ± 8.0	0.486
Female sex, n (%)	4840 (66.9)	211 (84.1)	<0.001
BMI (kg/m^2^), mean ± SD	22.8 ± 8.6	22.9 ± 8.5	0.8
Clinical Characteristics			
ASA score ≥ 3, n (%)	3747 (51.7)	118 (47)	0.062
Charlson Comorbidity Index, n (%)			0.002
Mild (1–2)	872 (12.1)	51 (20.3)	0.001
Moderate (3–4)	4354 (60.1)	131 (52.2)	0.1
Severe (≥5)	2011 (27.7)	69 (27.4)	0.5
Diabetes mellitus	1085 (15)	32 (13)	0.37
Congestive heart failure	144 (2)	5 (2)	1
COPD	333 (4.6)	11 (4.3)	1
Chronic kidney disease	289 (4)	7 (2.8)	0.35
Hip Fracture Type, n (%)			0.050
Intracapsular	2871 (39.7)	87 (34.7)	
Extracapsular	4366 (60.3)	164 (65.3)	

SD; Standard Deviation, ASA; American Society of Anaesthesiologists, BMI; Body Mass Index, COPD; Chronic Obstructive Pulmonary Disease.

**Table 2:** Comparison of primary and secondary outcomes.

Outcome	Isolated Hip Fracture (n = 7237)	Concomitant UE Fracture (n = 251)	*p*-Value
Primary Outcomes			
Length of stay (days), Mean ± SD	17.5 ± 17.7	20.2 ± 21.5	0.047
Mortality, n (%)			
30-day	303 (4.2)	8 (3.2)	0.439
90-day	586 (8.1)	17 (6.8)	0.167
1-year	1233 (17.0)	31 (12.4)	0.058
Secondary Outcomes			
Readmission, n (%)			
7-day	93 (1.3)	8 (3.2)	0.016
30-day	431 (6.0)	18 (7.2)	0.426
90-day	926 (12.8)	30 (12.0)	0.372
Complications at 1 year, n (%)			
Revision surgery	295 (4.1)	14 (5.6)	0.352
Any infection	1341 (18.5)	51 (20.3)	0.734
Surgical site infection	286 (4.0)	12 (4.8)	0.507
Non-surgical infection *	1055 (14.6)	39 (15.5)	0.864

* Non-surgical infections include urinary tract infections, pneumonia, and bacteremia. SD; Standard Deviation, UE; Upper Extremity.

**Table 3:** Outcomes by location of concomitant upper extremity fracture.

Outcome	Distal Radius (n = 114)	Proximal Humerus (n = 103)	Other UE Locations ‡ (n = 34)	Isolated Hip (n = 7237)	*p*-Value *
Length of stay (days) Mean ± SD	22.4 ± 22.5	20.1 ± 20.2	18.1 ± 20.8	17.5 ± 17.7	0.54
vs. Isolated (adjusted difference, 95% CI)	+4.0 days (0.1 to 8.9)	+2.4 days (−1.3 to 6.8)	−0.3 days (−5.3 to 6.7)	Reference	
*p*-value (vs. Isolated)	0.05	0.1	0.5		
Mortality					
30-day, n (%)	1 (0.9)	5 (4.9)	0 (0.0)		0.12
1-year, n (%)	9 (7.9)	16 (15.5)	6 (17.6)	1233 (17.0)	0.08
vs. Isolated (adjusted OR, 95% CI)	0.44 (0.23–0.85)	0.93 (0.55–1.57)	1.12 (0.48–2.64)	Reference	
*p*-value (vs. Isolated)	0.05	0.5	0.5		

‡ Other locations include olecranon (n = 20), humerus shaft (n = 5), radial head (n = 6), metacarpal (n = 3). * Distal radius vs. proximal humerus groups comparison. UE; Upper Extremity, SD; Standard Deviation, OR; Odds Ratio, CI; Confidence Interval.

**Table 4:** Outcomes by treatment approach for concomitant upper limb fracture.

Outcome	Non-Operative Treatment (n = 194)	Operative Treatment (n = 57)	*p*-Value
Length of stay (days) Mean ± SD	19.2 ± 19.9	26.2 ± 25.7	0.05
vs. Isolated * (adjusted)	+1.3 days (−2.1 to 5.2)	+8.6 days (3.8 to 14.2)	
1-year mortality, n (%)	28 (14.4)	3 (5.3)	0.07
Complications at 1 year, n (%)			
Revision surgery	6 (3.1)	8 (14.0)	0.01
vs. Isolated (adjusted OR)	0.79 (0.35–1.78)	4.02 (1.88–8.59)	
Any infection	36 (18.5)	10 (17.5)	1
Readmission (90-day)	21 (10.8)	6 (10.5)	1

SD; Standard Deviation, OR; Odds Ratio. * Compared to patients with isolated hip fractures.

**Table 5:** Outcomes by hip fracture type with and without concomitant fracture.

Hip Fracture Type	Group	LOS (Days) Mean ± SD	30-Day Mortality n (%)	1-Year Mortality n (%)
Intracapsular	Concomitant (n = 87)	21.2 ± 19.8	3 (3.4)	12 (13.8)
	Isolated (n = 2871)	17.3 ± 17.6	120 (4.2)	46 (16.3)
	Adjusted difference/OR	+5.5 days (1.2 to 10.8)	0.79 (0.25–2.50)	0.82 (0.45–1.49)
	*p*-value	0.05	0.5	0.5
Extracapsular	Concomitant (n = 164)	20.6 ± 22.4	5 (3.0)	19 (11.6)
	Isolated (n = 4366)	17.7 ± 17.8	183 (4.2)	764 (17.5)
	Adjusted difference/OR	+1.5 days (−1.8 to 5.1)	0.70 (0.29–1.69)	0.62 (0.39–0.99)
	*p*-value	0.1	0.5	0.05

SD; Standard Deviation, LOS; Length of stay.

## Data Availability

The data presented in this study are available on request from the corresponding author due to ethical restrictions.
